# Posterior dislocation of left hip joint with closed fracture of left acetabulum Judet-Letournel type posterior wall, femoral head fracture, management and follow up: A case report

**DOI:** 10.1016/j.ijscr.2020.04.009

**Published:** 2020-05-08

**Authors:** Ismail Hadisoebroto Dilogo, Uno Surgery Erwin, Andra Hendriarto

**Affiliations:** a Orthopaedic Trauma and Reconstruction, Department of Orthopaedic and Traumatology, Faculty of Medicine Universitas Indonesia, Dr. Cipto Mangunkusumo Hospital, Jakarta, Indonesia; b Department of Orthopaedic & Traumatology, Cipto Mangunkusumo National Central Hospital and Faculty of Medicine, Universitas Indonesia, Jalan Diponegoro No. 71, Central Jakarta, Jakarta 10430, Indonesia

**Keywords:** Hip, Dislocation, Acetabular, Fracture, Posterior, Case report

## Abstract

**Introduction:**

Traumatic dislocation of the hip is a very severe injury. Posterior hip dislocation can be associated with acetabular fracture, which can ultimately result in a higher incidence of complications.

**Case presentation:**

We present a case report of 26-year-old men with a motor vehicle accident and suffered a posterior hip dislocation on left hip joint and acetabular fracture with Judet-Letournel type posterior wall and femoral head fracture. This patient was performed closed reduction, however, due to dynamic instability of hip joint, internal Fixation through Kocher Langenback Approach and insertion of Herbert screws for femoral head and acetabular posterior wall fracture and interfragmentary screw for trochanteric osteotomy. The patient was followed up for 2 years and have a painless hip with full hip range of motion.

**Discussion:**

Posterior wall acetabular fractures sometimes occurs after posterior dislocation of the hip. Posterior wall fragment of the acetabular wall fracture can compromise the stability of the hip joint. The reduction can be maintained by closed means or by operative reduction if there is an obstacle to performed closed reduction or followed by sciatic nerve palsy after closed reduction. The complication of operative reduction is avascular necrosis which compromises the integrity of the femoral head and contributing to posttraumatic osteoarthritis.

**Conclusion:**

Careful examination of acetabular fracture in posterior hip dislocation is required and failed of closed reduction or instability of hip joint are indications to perform open reduction and internal fixation.

## Highlights

• Hip dislocation combines with femoral head fracture, and acetabular fracture is complex and rare case.
• The complication of operative reduction is avascular necrosis.
• Failed of closed reduction or instability of hip joint are indications to perform open reduction and internal fixation.

## 1 Introduction

Traumatic dislocation of the hip is a very severe injury. The hip has high stability, so to achieve splitting in the pelvis it needs a high force of at least 400 N. This dislocation is more often a result of a motor vehicle accident. The direction of the dislocation depends on the position of the lower limb at the time of the injury. Traumatic dislocations of the hip are divided into anterior and posterior types. Posterior dislocations are far more common than anterior dislocations. This dislocation is associated with forced adduction, internal rotation, and some degree of hip flexion. An acetabulum fracture can occur when femoral head transmits the force created by a direct blow to a trochanter mayor, knee, or leg. Acetabulum fractures can occur in this type of hip dislocation, but they rarely occur [1].

Hip dislocation can be associated with acetabular fracture, which can ultimately result in a higher incidence of complications. Complications can be caused by initial injury or by treatments such as avascular necrosis of the femoral head, degenerative osteoarthritis, and heterotrophic ossification [1,2]. We report a case of 26 years of men with posterior dislocation of the left hip and posterior wall acetabular fracture. The patient was managed in a national academic hospital setting. This work has been reported in line with the SCARE criteria [3].

## 2 Presentation of case

**Fig. 1:**
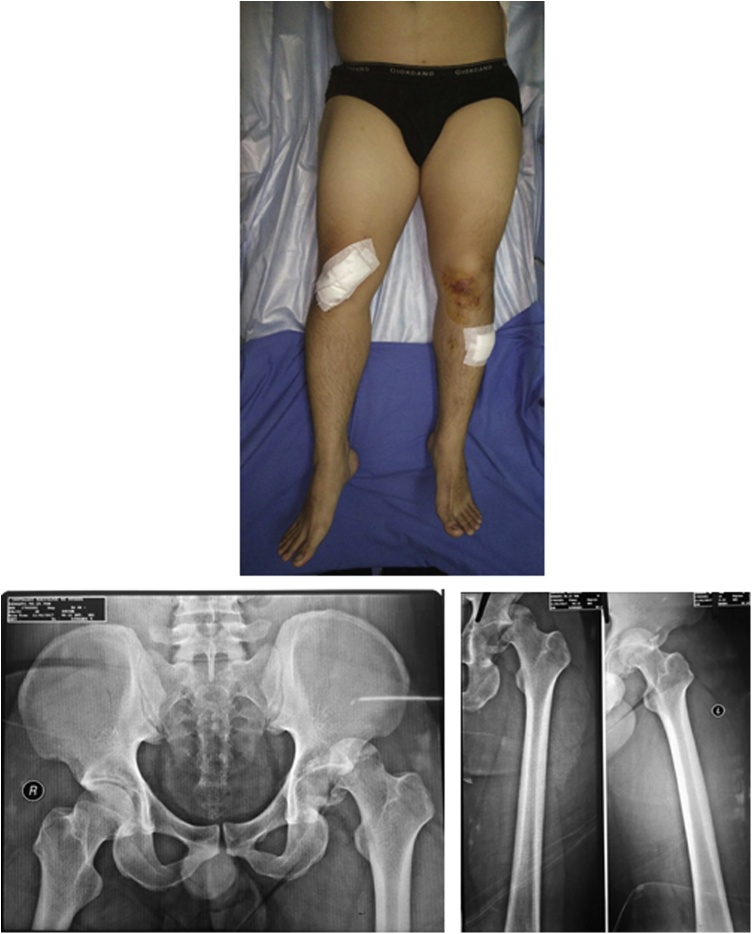
Local State of the Pelvic & Pelvic and Femoral X-Ray in The Emergency Room.

A 26-year-old male had a car accident 12 h ago, while he was on his way to work as a manager in a telecommunication company. The car hit the sidewalk in high speed, making the patient's left leg collided and detained. There were torn wounds on the left and right knees. The patient was taken to a private hospital in Jakarta, then the patient was referred to our institute by an ambulance after the wound were cleaned and covered. The patient did not experience any decrease of consciousness. In the initial assessment, pain was found in the left hip, intact neurovascular status, and no injuries that occurred together. The primary survey was clear. The patient denied any history of previous disease and history of previous surgery. The patient did not smoke and did not have any trouble walking before. There is a shortening, internal rotation, adduction, and shortening of the left hip and femur (Fig. 1). There is a pelvic pain with visual analog scale 3–4. Perfusion and distal sensory of extremities are good. Movement of the hip joint was limited by pain.

**Fig. 2:**
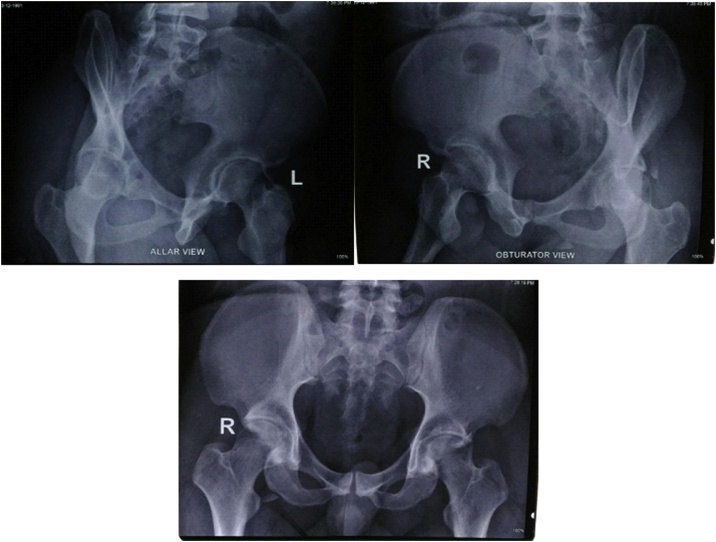
X-Ray After Closed Reduction; A) Allar View; B) Obturator View; C) Anteroposterior View.

**Fig. 3:**
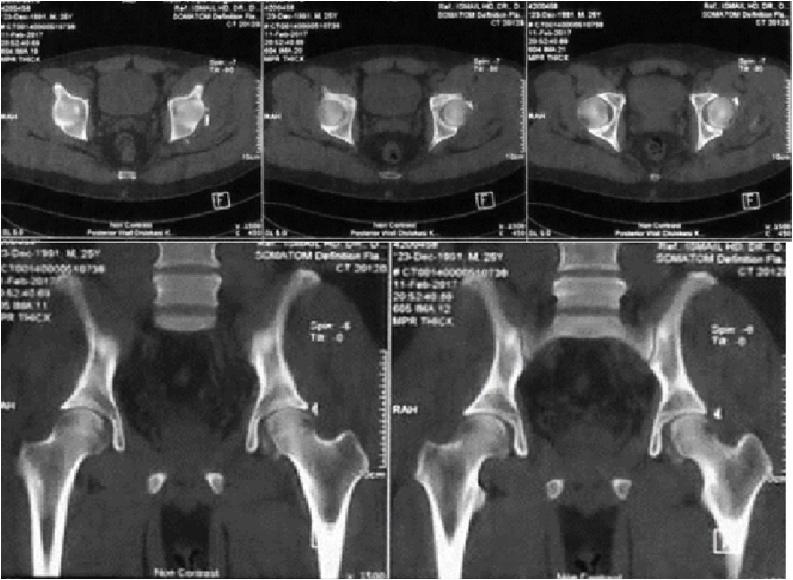
CT Scan: Evaluation of Fragment Size & Location.

The patient underwent investigations in the form of pelvic x-rays (Fig. 2) and femoral x-rays (Fig. 3), the results showed that the head of the left femur came out of the acetabulum and visible fractures on the posterior wall of the acetabulum. This patient was diagnosed with posterior dislocation of the left hip joint with a closed fracture of the left acetabulum Judet-Letournel type posterior wall. The management of this patient was an emergency closed reduction in general anesthesia with C arm in our institute.

**Fig. 4:**
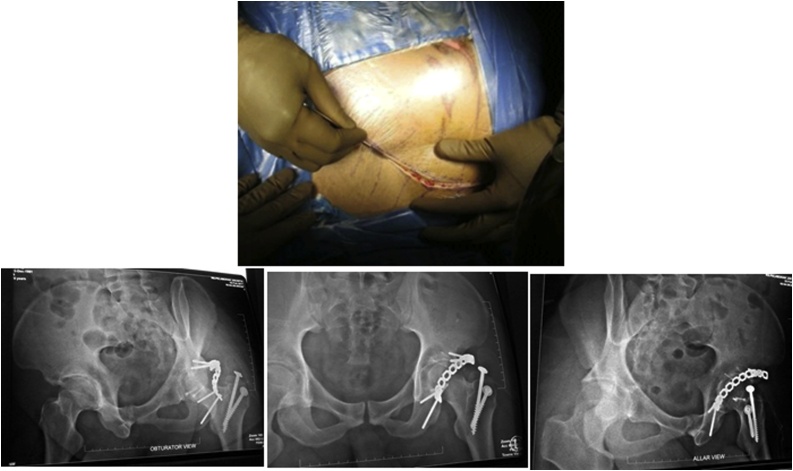
Internal Fixation through Kocher Langenback Approach & Postoperative X-ray After Surgery.

After closed reduction, the pelvic radiograph of the femoral head appeared to have returned to the acetabulum (Fig. 3). In addition to x-rays, a CT scan was also performed on the patient to evaluate the size of the fragment and location (Fig. 4).

**Fig. 5:**
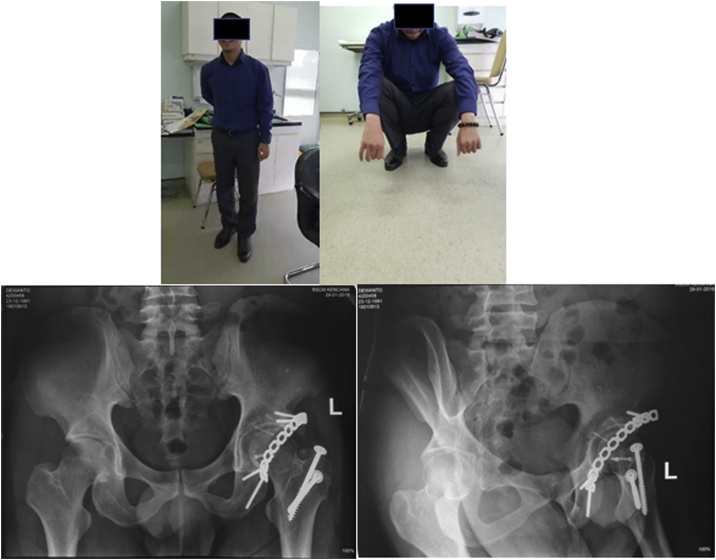
Patients were followed up for 2 years & X-ray after 2 years follow up.

ORIF was performed due to dynamic instability of the femoral head and posterior wall fracture Pipkin type 4. Kocher Langenback Approach with surgical hip dislocation technique in lateral decubitus position and the surgery conducted with the posterior kocher langenbach approach (Fig. 5). The procedure was performed by a trauma and reconstruction consultant and assisted by orthopedic resident.

After surgery, the patient was reevaluated with pelvic x-ray (Fig. 4). There were 2 Herbert screws for femoral head fracture, 2 Herbert screws and Buttress Plate at the posterior wall, and Interfragmentary screw for trochanteric osteotomy.

The patient were followed up for 2 years, patient had lost 23 kg of body weight, painless hip, even on mountain climbing, full hip range of motion (Fig. 5). On X-Ray (Fig. 5), there was a solid union at trochanteric osteotomy site, no sign of avascular necrosis, no arthritic changes on the hip joint.

## 3 Discussion

The hip joint is one of the most stable joints in the body due to its ball and socket architecture and tight ligament and muscular structures. The hip joint is relatively stable, so excessive force is needed to make hip joint dislocate. Dislocation of the hip is usually a result of high-energy trauma. Association between hip dislocation and posterior acetabular wall fracture is very frequent but the combination with intertrochanteric fracture is very rare [4].

There are three types of acetabular fracture; column fractures, transverse fractures, and wall fractures. Acetabular wall fractures frequently involve the posterior wall but occasionally involve the anterior wall. It is important to note that wall fractures are unique from the two other fracture patterns due to the loadbearing mechanism of the acetabulum may not be disrupted [2]. Posterior dislocations of the hip are more frequent than anterior dislocations. These dislocations correlate with forced adduction, internal rotation, and some degree of flexion of the hip. Fractures of the acetabulum occur when the femoral head transmits forces created by a direct blow to the greater trochanter, knee, or foot [1].

The anatomic and radiographic classification proposed by Judet et al. has been shown to be clinically useful because it outlines a plan of treatment. Based on a standard radiographic examination (AP and Judet oblique views) and most recently on CT scan studies this classification constitutes the first step in surgical decision making. According to this system, acetabular fractures are divided into elementary and associated fractures. In general, posterior dislocations of the hip with acetabular fractures must be addressed via a posterior *approach*.

Elementary fractures consist of five types: 1) Posterior wall; 2) Posterior column; 3) Anterior wall; 4) Anterior column; 5) Transverse, whereas associated fractures consist of five types: 1) T shaped; 2) Posterior wall plus posterior column; 3) Posterior wall plus transverse; 4) Anterior column or wall plus posterior hemitransverse; 5) Both columns [1].

The typical patient with a posterior dislocation of the hip presents with adduction, internal rotation, flexion, and shortening of the limb. A contusion or excoriation over the knee or greater trochanter, or an adducted femur should alert the surgeon to the possibility of a hip dislocation. A routine anteroposterior (AP) radiograph of the pelvis is mandatory in all patients sustaining multiple trauma. In posterior fracture-dislocations of the hip, the AP radiograph of the pelvis usually will display a small femoral head above the acetabular dome and different patterns of acetabular fractures. Posterior wall fractures are the most common pattern, accounting for 18%–33% of all acetabular fractures. Computed tomography (CT) scans obtained before reduction are helpful in the evaluation of and decision making about the injured hip. Magnetic resonance imaging has been proven to have a limited role in the diagnosis and assessment of posterior fracture-dislocation of the hip. However, MRI can effectively identify nondisplaced fractures that are not apparent on axial CT scans [1,5].

Reduction usually is accomplished by closed means unless there is an indication to performed open reduction. In general, a primary operative reduction is recommended if there is an obstacle to closed reduction, such as intraarticular loose bodies and soft tissue interposition within the joint space, or there is sciatic nerve palsy after closed reduction. Primary open reduction in these cases eliminates added trauma, precluding complications such fracture of the femoral neck and early onset of arthritis [1].

Dislocations of the hip with open acetabular fractures are associated with a high mortality rate and must be irrigated and debrided promptly followed by reduction and stabilization. In patients with open fractures of the pelvis, the infection rate may be as high as 30%, and the mortality rate may be as high as 50%. These patients require extensive debridement of all dead tissue, reduction of the hip dislocation, and, if possible, internal fixation of the open fractures within 6–12 h after injury [1,6].

Posterior wall acetabular fractures are the most frequent pattern found after a posterior dislocation of the hip. When the fragment involves 25%–50% of the acetabulum (transitional fragments), the stability depends on the integrity of the joint capsule. If the capsule is intact, closed treatment of the acetabular fracture can be performed. Unfortunately, dislocations of the hip occur almost exclusively after the capsule is torn, becoming incompetent in its role for stabilizing the hip. It is thought that if there are dislocation and a posterior wall fragment, the wall should be fixed because of the instability secondary to the dislocation, regardless of the size of the posterior wall fragment [1,6]. Anatomic reduction of the fracture is the most important clinical outcome in acetabular surgery [7].

The most threatening complications of these cases are avascular necrosis (AVN) of the femoral head. Open reduction procedures conducted for hip joint dislocation raise the risk of AVN and asymptomatic until 1-year follow of the patient. Avascular necrosis (AVN) was found in patients which had been treated using an extended iliofemoral approach and 20 through a Kocher-Langenbeck approach, giving a proportional occurrence of heterotopic bone of 38% and 20%, respectively for these incisions [4,5]. If avascular necrosis of the femoral head occurs, total hip arthroplasty can be performed to the patient and have a good outcome to reduce morbidity and risk of revision surgery [8]. Other complication which can appear after hip dislocation and acetabular fracture is posttraumatic arthritis. Posttraumatic arthritis have a correlation in severity of injury [9].

Case published by Jamshidi et al. explain the case report of Posterior Hip Fracture -Dislocation Associated with ipsilateral intertrochanteric fracture. This case involve 26 years old patient after high energy dashboard injury and performed reduction and surgical reconstruction 24 h after injury. Patient was inserted reconstructive plate similar with our patient, with additional dynamic hip screw (DHS) for the intertrochanteric fracture [4].

Other case, published by Qi et al., explained the posterior dislocation of the hip followed by a bilateral femoral fracture with acetabular fracture in one side of the pelvis. The trauma caused by high energy trauma in car accident. It was revealed that similar to this case, there is a posterior hip dislocation viewed on X-Ray and CT revealed that there is a right acetabular fracture simultaneously with posterior hip dislocation. Differs with our patient, the patient is failed to be performed closed reduction and then performed open reduction with administration of internal fixation through Kocher-Langenbeck approach, however in our case the patient is successful to be performed closed reduction, but hip instability is required this joint to do the open reduction and internal fixation procedure [10].

## 4 Conclusion

This case is important because the combination of hip dislocation, femoral head fracture, and acetabular fracture is complex and rare case. It is important to exclude posterior wall acetabular fracture in patient with clinical diagnosis of posterior hip dislocation. Failed of closed reduction and instability of hip joint is indication to perform open reduction and internal fixation with reconstructive plate.

## Declaration of Competing Interest

The authors declare that there is no conflict of interest regarding the publication of this article.

## Sources of funding

The authors received no financial support for the research, authorship, and/or publication of this article.

## Ethical approval

The ethical approval was not required for this case report.

## Consent

Informed consent had been obtained from the patient before the manuscript was written.

## Author contribution

Ismail Hadisoebroto Dilogo: Concept of the study, data collection & interpretation, and writing the paper.

Uno Surgery Erwin: Data collection, data interpretation and writing the paper.

Andra Hendriarto: data collection & interpretation and writing the paper.

## Registration of research studies

NA.

## Guarantor

Ismail Hadisoebroto Dilogo.

## Provenance and peer review

Not commissioned, externally peer reviewed.
